# Transforming and integrating STI surveillance to enhance global advocacy and investment in STI control

**DOI:** 10.1002/jia2.25361

**Published:** 2019-08-30

**Authors:** Melanie M Taylor, Teodora EC Wi

**Affiliations:** ^1^ Department of Reproductive Health and Research World Health Organization Geneva Switzerland; ^2^ United States Centers for Disease Control and Prevention Atlanta GA USA

**Keywords:** sexually transmitted infections, surveillance, gonorrhoea, syphilis, chlamydia, trichomoniasis

`Sexually transmitted infections (STI) exact an astounding yet preventable toll on the health and lives of men and women worldwide. The World Health Organization (WHO) estimated 376 million new curable STI occurred in 2016, including chlamydia (127 million), gonorrhoea (87 million), syphilis (6.3 million) and trichomoniasis (156 million) 1. More than 500 million people were estimated to have genital infections with herpes simplex virus (HSV‐1 or HSV‐2) in 2012 2. Approximately 290 million women were estimated to have a human papillomavirus (HPV) infection in 2007 3. These infections have predictably serious complications for the men and women infected and their new‐born infants. More than 500,000 incident cervical cancer cases, caused by HPV occurred in 2018, with a greater than 50% mortality rate 4. For 2016, WHO estimated 988,000 pregnant women were infected with syphilis resulting in 660,000 congenital syphilis cases of which 350,000 were adverse birth outcomes including stillbirth and neonatal death 5. Additional STIs such as viral hepatitis, *Mycoplasma genitalium* infection, and lymphogranuloma venereum add further weight to these estimates 6, 7. Newly emerging viral pathogens Ebola and Zika have gained prominent attention as they are each sexually transmitted. 8, 9

STI have been associated with increased HIV transmission 10, 11. Yet while remarkable progress has been made in reducing HIV transmission and improving lives of patients with anti‐retroviral therapy (ART), STI incidence is high and increasing in many regions 1 (Figure 1). Although antiretroviral pre‐exposure prophylaxis (PrEP) is associated with reduced HIV transmission, STI incidence tends to be high among PrEP patients, as well among persons living with HIV and other vulnerable populations 12, 13, 14. The biological and behavioural links between HIV and STIs suggest opportunities for improving STI control and surveillance through existing HIV prevention, testing, and treatment services.

**Figure 1:**
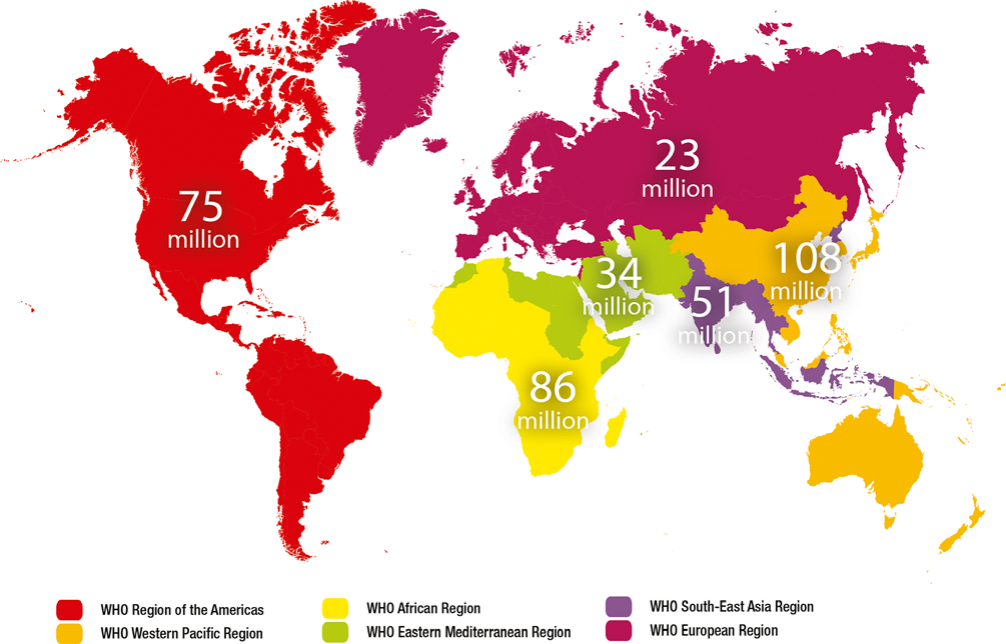
Estimated new cases of curable sexually transmitted infections (gonorrhoea, chlamydia, syphilis and trichomoniasis) by WHO region, 2016 1.

In 2016, three linked WHO strategies for HIV, hepatitis and STIs were endorsed by the World Health Assembly 6, 7, 15. Each of these strategies called for integration across fields of surveillance and service delivery for these three infection groups.

The WHO strategy on STIs (2016 to 2021) identified four targets for 2030 7.

- 90% reduction in *Treponema pallidum* incidence globally (based on the 2018 global baseline).
- 90% reduction in *Neisseria gonorrhoeae* incidence globally (based on the 2018 global baseline).
- ≤50 cases of congenital syphilis per 100,000 live births in 80% of countries
- Sustain 90% national coverage and at least 80% in every district (or equivalent administrative unit) in countries with the human papillomavirus vaccine in their national immunization programme.

Robust national‐level strategic information systems that incorporate STI case reporting, prevalence surveys, assessment of the aetiology of STI syndromes, and monitoring for antimicrobial resistance to gonorrhoea are needed to guide programming and clinical service delivery 7, 16 (Box 1). As reported in this issue by Wi et al., most countries lack the basic capacity to diagnose and treat STIs let alone implement surveillance 17. Yet potential stakeholders must first recognize the prevalence and impact of these infections from reliable surveillance data. A vicious cycle of limited STI surveillance and narrow STI program response continues in most resource limited settings. Countries need strong strategic information systems that incorporate STIs to inform and help target prevention and treatment efforts, to rally political commitment, and build a strong national investment case. It is essential for countries to know their STI epidemics and to know the recommended responses in order that up to date, accurate information can guide national programming.

Box 1 **WHO National and Global Sexually Transmitted Infection Surveillance Priorities for Action 7**

**Priority actions for countries**

- **Strengthen and integrate sexually transmitted infection surveillance into the national health information system** as a part of health system strengthening, using standardized indicators and methodologies *as guided by WHO; ensure that data collection methods yield high‐quality information, meet ethical standards, and do not pose risks for communities or the health care workers involved*.
- **Increase the “granularity” of data** including through: enhanced sexually transmitted infection‐related disaggregated data collection based on different stratifiers that include age, sex, population and location*; involve affected communities and specific populations to achieve high‐quality data and analysis*.
- **Identify specific populations** who are most at risk for sexually transmitted infections and places where most of the transmission is occurring; *establish mechanisms to promote the participation of affected communities;* conduct routine case reporting and periodic prevalence assessments of core sexually transmitted infections to assess the magnitude of the sexually transmitted infection problem in target populations, including by disaggregating the data*; describe the sexually transmitted infection epidemics and measure the impact in terms of sequelae and cost*.
- **Include data on the risk factors and determinants of sexually transmitted infections** in order to understand and address these determinants. *Include a focus on pre‐exposure prophylaxis as appropriate. Use both standard and innovative participatory survey methodologies to develop accurate estimates of key population sizes and detailed understandings of subnational epidemics; integrate biological surveillance with other programmes, such as a behavioural surveillance survey in the HIV files – include contact tracing and treatment of partners*.
- **Strengthen national laboratory capacity** through quality assurance and the introduction of point‐of‐care diagnostics to ensure routine monitoring of sexually transmitted infections and antimicrobial resistance to *Neisseria gonorrhoeae*.

**Priority actions for WHO**

- **Provide global leadership and assistance to countries** in strengthening sexually transmitted infection surveillance and in using standard methodologies for such surveillance and estimation of the burden and impact; support the development of strategic information systems and sexually transmitted infection epidemics and response mapping, including the analysis of disaggregated data for monitoring inequities; support countries in strengthening case reporting, prevalence assessment, aetiologic assessment and antimicrobial resistance monitoring; strengthen global systems for collecting and sharing national surveillance data on sexually transmitted infections, including disaggregated data and analysis for monitoring equity.
- **Provide guidance on the collection and analysis of disaggregated data** based on different stratifiers and the involvement of affected communities and specific populations, including key populations for HIV, in efforts to obtain high‐quality data and achieve high‐quality analysis; use internationally endorsed methods for estimating the sizes of key populations for HIV and on setting programme targets for services for key populations for HIV.
- **Ensure linkages** of some components of sexually transmitted infection surveillance to existing mechanisms including HIV and antimicrobial resistance surveillance.

WHO has developed frameworks, targets and priority actions for STI surveillance at national and global levels 7, 16, 18 (Box 1). Global strategic information systems like the UNAIDS Global AIDS Monitoring system (GAM) 19 have helped to align national‐level reporting of key STI indicators related to syphilis and gonorrhoea alongside those of HIV, but reported data are incomplete and many countries are challenged to collect verifiable data. WHO has supported the development of freely available modelling tools such as Spectrum STI 20 and the WHO congenital syphilis estimation tool 21 to allow the use of country‐reported data to conduct national‐level analysis of incidence and prevalence trends. WHO conducts global surveillance for antimicrobial resistance in gonorrhoea, which captures proportions of resistant organisms from nearly 60 countries 22.

High‐income and low‐middle income countries with STI surveillance systems frequently rely on case reporting of STI cases or STI syndromes to estimate national incidence 23, 24, 25, 26. Case reporting drastically underestimates the burden of STIs due to the asymptomatic nature of infection, limited access to care for those with symptoms, and limited provider reporting 18. For these reasons, case reporting alone would not be a reliable measure of national STI burden. National, regional and global incidence and prevalence can be derived from longitudinal STI prevalence surveys using standard methods 18. STI prevalence surveys among general and high‐risk population groups of men and women can be conducted as part of population‐based health surveys such as those done for HIV, or in association with other health surveys or health services such as HIV screening and prevention (PrEP), maternal, reproductive, adolescent and child health services and military, work‐related or school‐based health screening. As STIs are not equally distributed among sexually active populations and a disproportionally higher burden of the STI/HIV epidemic occurs among certain key population sub‐groups, such as men who have sex with men and sex workers, specialized surveillance and culturally tailored programmes to address STIs among these populations are warranted. Routine STI prevalence assessments can identify key populations that can benefit from the implementation of effective STI interventions and further provide evidence of their impact. Global, regional and national estimates of STIs suffer from limited prevalence surveys among general populations, particularly among men 1.

It is evident from recent global and regional estimates of STIs that the necessary stakeholder support, advocacy and investment – both national and international – to support STI programme and surveillance efforts has not been realized. While the burden of prevalent and incident STI cases increases, advocacy for control of these infections has waned. Transforming and strengthening STI surveillance and clinical services can serve as a cornerstone for advocacy and investment in STI prevention and control. Alignment of STI control programmes alongside HIV and hepatitis prevention through linked WHO strategies has offered frameworks for integration yet clinical services and surveillance of STIs continue to lag behind 27.

As part of a transformation process taking place at WHO, set in motion by the Director General in 2018, the global STI surveillance and STI programme support activities will be moved from the WHO Department of Reproductive Health and Research (WHO RHR) to the Department of HIV and Hepatitis, to be duly renamed the WHO Department of HIV, Hepatitis and STIs. STI research will remain with (WHO RHR) ensuring that research continues to inform STI programming. The move of the STI programme will set an example at the global level of the opportunity to integrate these surveillance and country support activities recognizing similar modes of transmission, populations at risk and currently existing health care platforms. This transition is expected to herald a renewed global focus on the importance of STIs as indicators of HIV and hepatitis risk and as opportunities for prevention and control of all STIs while ensuring continued inclusion within the broader framework of sexual and reproductive health and rights 28.

## Competing interests

The authors have no competing interests to declare.

## Authors’ contributions

MT and TW conceived of the paper and provided content and references. MT drafted the paper. MT and TW reviewed and revised drafts prior to submission

## References

[1] Rowley J , Vander Hoorn S , Korenromp E , Low N , Unemo M , Abu‐Raddad LJ , et al. Global and Regional Estimates of the Prevalence and Incidence of Four Curable Sexually Transmitted Infections in 2016. WHO Bulletin. June 2019 [cited 2019 June 12]. Available at: https://www.who.int/bulletin/online_first/BLT.18.228486.pdf

[2] Looker KJ , Magaret AS , Turner KM , Vickerman P , Gottlieb SL , Newman LM . Global estimates of prevalent and incident herpes simplex virus type 2 infections in 2012. PLoS ONE. 2015;10(1):e114989. 10.1371/journal.pone.0114989 PMC4301914 25608026

[3] de Sanjosé S , Diaz M , Castellsagué X , Clifford G , Bruni L , Muñoz N , et al. Worldwide prevalence and genotype distribution of cervical human papillomavirus DNA in women with normal cytology: a meta‐analysis. Lancet Infect Dis. 2007;7(7):453–9. 17597569 10.1016/S1473-3099(07)70158-5

[4] Bray F , Ferlay J , Soerjomataram I , Siegel RL , Torre LA , Ahmedin J . Global Cancer Statistics 2018: GLOBOCAN Estimates of Incidence and Mortality Worldwide for 36 Cancers in 185 Countries. CA Cancer J Clin. 2018;68:394–424. 30207593 10.3322/caac.21492

[5] Korenromp EL , Rowley J , Alonso M , Mello MB , Wijesooriya NS , Mahiané SG . Global burden of maternal and congenital syphilis and associated adverse birth outcomes—Estimates for 2016 and progress since 2012. PLoS ONE. 2019;14(2):e0211720. 30811406 10.1371/journal.pone.0211720 PMC6392238

[6] World Health Organization . Global Health Sector Strategy on viral hepatitis, 2016–2021. [cited 2019 June 20]. Available at: https://www.who.int/hepatitis/strategy2016-2021/ghss-hep/en/

[7] World Health Organization . Global Health Sector Strategy on Sexually Transmitted Infections, 2016–2021. [cited 2019 June 20]. Available at: https://www.who.int/reproductivehealth/publications/rtis/ghss-stis/en/

[8] World Health Organization . Interim advice on the sexual transmission of the Ebola virus disease. [cited 2019 Jun 17]. Available at: https://www.who.int/reproductivehealth/topics/rtis/ebola-virus-semen/en/

[9] World Health Organization . Guidelines for the prevention of sexual transmission of Zika virus: Executive summary. [cited 2019 Jun 17]. Available at: https://www.who.int/reproductivehealth/zika/prevention-guidelines-sexual-transmission-summary/en/

[10] Wasserheit JN . Epidemiologic synergy: interrelationships between HIV and other STDs. Sex Transm Dis. 1992;19:61–7. 1595015

[11] Cohen MS . HIV and sexually transmitted diseases: lethal synergy. Top HIV Med. 2004;12(4):104–7. 15516707

[12] Kojima N , Davey DJ , Klausner JD . Pre‐exposure prophylaxis for HIV infection and new sexually transmitted infections among men who have sex with men. AIDS. 2016;30(14):2251–2. 27314179 10.1097/QAD.0000000000001185

[13] Traeger MW , Schroeder SE , Wright EJ , Hellard ME , Cornelisse VJ , Doyle JS , et al. Effects of pre‐exposure prophylaxis for the prevention of human immunodeficiency virus infection on sexual risk behavior in men who have sex with men: a systematic review and meta‐analysis. Clin Infect Dis. 2018;67(5):676–86. 29509889 10.1093/cid/ciy182

[14] Delany‐Moretlwe S , Chersich M , Harvey S , Stangl A , Baron D , Columbini M , et al. Empowerment clubs did not increase PrEP continuation among adolescent girls and young women in South Africa and Tanzania‐Results from the EMPOWER randomised trial. Conference paper presented at the 22nd International AIDS Conference, Amsterdam, Netherlands. J Int AIDS Soc. 2018;21(S6). 10.1002/jia2.25148

[15] World Health Organization . Global health sector strategy on HIV, 2016‐2021. [cited 2019 June 20]. Available at: https://www.who.int/hiv/strategy2016-2021/ghss-hiv/en/

[16] World Health Organization . WHO tool for STI Surveillance Strengthening. [cited 2019 June 22]. Available at: https://www.who.int/reproductivehealth/publications/rtis/sti-surveillance/en/

[17] Wi TEC , Ndowa FJ , Ferreyra C , Kelly‐Cirino C , Taylor MM , Toskin I , et al. Diagnosing sexually transmitted infections in resource‐constrained settings: challenges and ways forward. J Int AIDS Soc. 2019;22(S6):e25343. 31468679 10.1002/jia2.25343 PMC6715950

[18] World Health Organization . Standard Protocol to assess prevalence of GC and CT in pregnant women. [cited 2019 June 25]. Available at: https://www.who.int/reproductivehealth/publications/rtis/gonorrhoea-chlamydia-among-pregnant-women/en/

[19] UNAIDS . Global AIDS Monitoring 2019 2018 GUIDANCE Indicators for monitoring the 2016 Political Declaration on Ending AIDS. 2019 [cited 2016 July 1]. Available at: https://www.unaids.org/en/resources/documents/2018/Global-AIDS-Monitoring

[20] Avenir Health . Spectrum STI Modeling Tool. [cited 2019 Jun 17]. Available at: https://www.avenirhealth.org/software-spectrum.php

[21] World Health Organization . Congenital Syphilis Estimation Tool. [cited 2019 Jun 20]. Available at: https://www.who.int/reproductivehealth/congenital-syphilis/surveillance/en/

[22] Wi T , Lahra M , Ndowa F , Bala M , Dillon J , Ramon‐Pardo P , et al. Antimicrobial resistance in *Neisseria gonorrhoeae*: Global surveillance and a call for international collaborative action. PLoS Med. 2017;14(7):e1002344. 28686231 10.1371/journal.pmed.1002344 PMC5501266

[23] Centers for Disease Control and Prevention . Sexually transmitted disease surveillance 2016. Atlanta, GA: U.S. Department of Health and Human Services; 2017 [cited 2019 June 28]. Available at: https://www.cdc.gov/std/stats17/2017-STD-Surveillance-Report_CDC-clearance-9.10.18.pdf

[24] Public Health England, Health Protection Report. Volume 12, Number 20, 8 June 2018 [cited 2019 June 28]. Available at: https://www.gov.uk/government/publications/health-protection-report-volume-12-2018

[25] Report on global sexually transmitted infection surveillance, 2018. Geneva: World Health Organization; 2018 [cited 2019 June 20]. Licence: CC BY‐NC‐SA 3.0 IGO]. Available at: https://www.who.int/reproductivehealth/publications/stis-surveillance-2018/en/

[26] European Centre for Disease Prevention and Control . Syphilis and congenital syphilis in Europe – A review of epidemiological trends (2007‐2018) and options for response. Stockholm: ECDC; 2019 https://ecdc.europa.eu/sites/portal/files/documents/Syphilis-and-congenital-syphilis-in-Europe.pdf. Accessed on July 16, 2019

[27] Progress report on HIV, viral hepatitis and sexually transmitted infections 2019. Accountability for the global health sector strategies, 2016–2021. Geneva: World Health Organization; 2019. (WHO/CDS/HIV/19.7). Licence: CC BY‐NC‐SA 3.0 IGO.

[28] World Health Organization . The Global Strategy for Women's, Children's and Adolescents’ Health, 2016‐2030 [cited 2019 June 22]. Available at: https://www.who.int/life-course/partners/global-strategy/en/ 10.2471/BLT.16.174714 PMC4850547 27147756

